# Non-invasive detection of endometrial cancer by DNA methylation analysis in urine

**DOI:** 10.1186/s13148-020-00958-7

**Published:** 2020-11-03

**Authors:** Rianne van den Helder, Birgit M. M. Wever, Nienke E. van Trommel, Annina P. van Splunter, Constantijne H. Mom, Jenneke C. Kasius, Maaike C. G. Bleeker, Renske D. M. Steenbergen

**Affiliations:** 1 Amsterdam UMC, Vrije Universiteit Amsterdam, Department of Pathology, Cancer Center Amsterdam, De Boelelaan 1117, Amsterdam, The Netherlands; 2 Antoni van Leeuwenhoek/Netherlands Cancer Institute, Center of Gynecologic Oncology Amsterdam, Department of Gynecologic Oncology, Amsterdam, The Netherlands; 3 Amsterdam UMC, University of Amsterdam, Center of Gynecologic Oncology Amsterdam, Department of Gynecologic Oncology, Cancer Center Amsterdam, Meibergdreef 9, Amsterdam, The Netherlands

**Keywords:** Endometrial cancer, Cancer detection, Biomarkers, DNA methylation, Urine, Non-invasive, Liquid biopsy

## Abstract

**Background:**

The incidence of endometrial cancer is rising, and current diagnostics often require invasive biopsy procedures. Urine may offer an alternative sample type, which is easily accessible and allows repetitive self-sampling at home. Here, we set out to investigate the feasibility of endometrial cancer detection in urine using DNA methylation analysis.

**Results:**

Urine samples of endometrial cancer patients (*n* = 42) and healthy controls (*n* = 46) were separated into three fractions (full void urine, urine sediment, and urine supernatant) and tested for three DNA methylation markers (*GHSR*, *SST*, *ZIC1*). Strong to very strong correlations (*r* = 0.77–0.92) were found amongst the different urine fractions. All DNA methylation markers showed increased methylation levels in patients as compared to controls, in all urine fractions. The highest diagnostic potential for endometrial cancer detection in urine was found in full void urine, with area under the receiver operating characteristic curve values ranging from 0.86 to 0.95.

**Conclusions:**

This feasibility study demonstrates, for the first time, that DNA methylation analysis in urine could provide a non-invasive alternative for the detection of endometrial cancer. Further investigation is warranted to validate its clinical usefulness. Potential applications of this diagnostic approach include the screening of asymptomatic women, triaging women with postmenopausal bleeding symptoms, and monitoring women with increased endometrial cancer risk.

## Background

Endometrial cancer (EC) is the most common gynecological cancer in developed countries and the sixth most common cancer worldwide [1]. Its incidence is rising globally [2] with over 380,000 new cases and 89,929 deaths reported in 2018 [3]. The increasing incidence of EC is partly attributable to the rise in the prevalence of risk factors associated with EC development, like obesity [4, 5].

Despite the rising incidence of EC and proven value of early diagnosis, no screening program for EC exists [6, 7]. In addition, if EC is suspected, invasive biopsy procedures remain necessary in routine clinical practice to detect EC in symptomatic women. Besides, the opportunity to detect EC in asymptomatic women by cytological evaluation of cervical scrapes during cervical cancer screening programs will be missed by the transition towards a primary high-risk human papillomavirus screening approach in many countries.

Hence, there is a need to detect EC using less invasive sampling methods, combined with the analysis of cancer-specific markers [6]. One of the emerging biomarkers for early cancer detection is DNA methylation, which involves the addition of a methyl group to a cytosine-guanine dinucleotide (CpG). Altered DNA methylation is a common epigenetic event that occurs during the early stages of carcinogenesis of many cancer types, including EC, and has been linked to gene silencing of tumor suppressor genes. Testing for elevated DNA methylation levels of specific genes is promising in early cancer detection [8].

Previous studies have shown that aberrant EC-specific DNA methylation signatures can be measured in various minimally invasive sample types, including cervical scrapes [9–12], endometrial brushes [13], vaginal swabs [14, 15] and vaginal tampons [16, 17]. The ability to detect EC in cervicovaginal samples implicates shedding of endometrial cells and cell fragments into the lower genital tract, and, potentially, also into the urine. Apart from cellular tumor DNA, tumor-derived DNA can be released into the bloodstream as cell free DNA (cfDNA) and pass to the urine by filtration through transrenal excretion [18, 19]. The suitability of EC detection in urine has been supported by the presence of EC-specific micro-RNAs in urine [20, 21]. The measurement of DNA methylation markers in urine has been proven useful for the detection of cervical cancer [22, 23], as well as other cancers, including bladder [24–27], lung [28], and prostate cancer [29–32]. However, to the best of our knowledge, no such approach has been investigated for the detection of EC.

The majority of DNA methylation markers that hold promise for EC detection have been derived from studies on EC, but also markers developed for cervical cancer detection showed potential diagnostic relevance for EC detection [33]. We considered the markers *GHSR, SST* and *ZIC1* as interesting candidates to evaluate the detection of EC in the urine by DNA methylation marker testing, based on our previous studies on urinary methylation markers and their diagnostic marker potential for different cancer types [22, 23, 25, 34].

This study investigates the feasibility of DNA methylation analysis in different urine fractions for the detection of EC. DNA methylation of genes *GHSR*, *SST*, and *ZIC1* was analyzed in full void urine, urine sediment and urine supernatant samples of women with various types, histological grades and stages of EC and a healthy control group to determine the most optimal urine fraction and applicability of these genes for the detection of EC in the urine.

## Results

### Patient characteristics

A total of 42 EC patients and 46 healthy controls were enrolled in this study. An overview of clinical characteristics is displayed in Table 1.

**Table 1:** Patient characteristics

Healthy controls		
* n*	46	
Age: median	56	
Age: min–max	45–82	
Endometrial cancer cases		
* n*	42	
Age: median	66	
Age: min–max	40–86	
Histology	*n*	%
Endometrioid	23	54.8
Grade 1	8	
Grade 2	7	
Grade 3	8	
Serous	11	26.2
Carcinosarcoma	4	9.5
Clear cell	1	2.4
Mixed^a^	3	7.1
FIGO stage	*n*	%
I	27	64.3
II	3	7.1
III	7	16.7
IV	5	11.9

^a^Patients with endometrial carcinomas of mixed subtypes included two mixed clear cell and endometrioid carcinomas and one mixed serous and carcinosarcoma

### DNA quality of urine fractions

To select the most suitable urine fraction for DNA methylation analysis, the quality of DNA isolated from paired full void urine, urine sediment, and urine supernatant samples was first assessed by comparing the quantification cycle (Cq) values of the reference gene *ACTB* (Table 2). While the Cq values of *ACTB* were nearly identical in full void urine samples (24.7) and urine sediments (24.8), they were significantly higher (*p* < 0.001) in urine supernatant samples (26.1). Of note, amongst the different fractions, none of the samples tested invalid in urine sediment, as compared to two in both full void urine and urine supernatant samples.

**Table 2:** DNA quality characteristics of paired urine fractions of controls and EC patients (*n* = 76)

	Full void urine		Urine sediment		Urine supernatant	
	Median Cq	Invalid (%)	Median Cq	Invalid (%)	Median Cq	Invalid (%)
*ACTB*	24.7	2 (2.6)	24.8	0 (0.0)	26.1	2 (2.6)

EC: endometrial cancer

Invalid (%): invalid for methylation analysis based on a Cq value for *ACTB* ≥ 32

### Comparison of DNA methylation analysis in different urine fractions

Subsequently, the DNA methylation levels of *GHSR, SST*, and *ZIC1* were compared among paired urine fractions to determine the correlation between the different urine components. For all markers, a strong to very strong (*r* ≥ 0.77–0.92) correlation was found between different urine fractions of women with EC (Table 3).

**Table 3:** Correlation of methylation markers between paired urine fractions from EC patients (*n* = 40)

	Full void urine versus urine sediment	Full void urine versus urine supernatant	Urine sediment versus urine supernatant
*GHSR*	0.85	0.92	0.89
*SST*	0.78	0.91	0.74
*ZIC1*	0.87	0.90	0.77

EC: endometrial cancer

Spearman’s correlation coefficient (*r*) was calculated based on the log2-transformed Cq ratios. *r* = 0.40–0.59 moderate correlation, *r* = 0.60–0.79 strong correlation, *r* = 0.80–1.00 very strong correlation

### DNA methylation as diagnostic marker for EC detection in each urine fraction

**Fig. 1:**
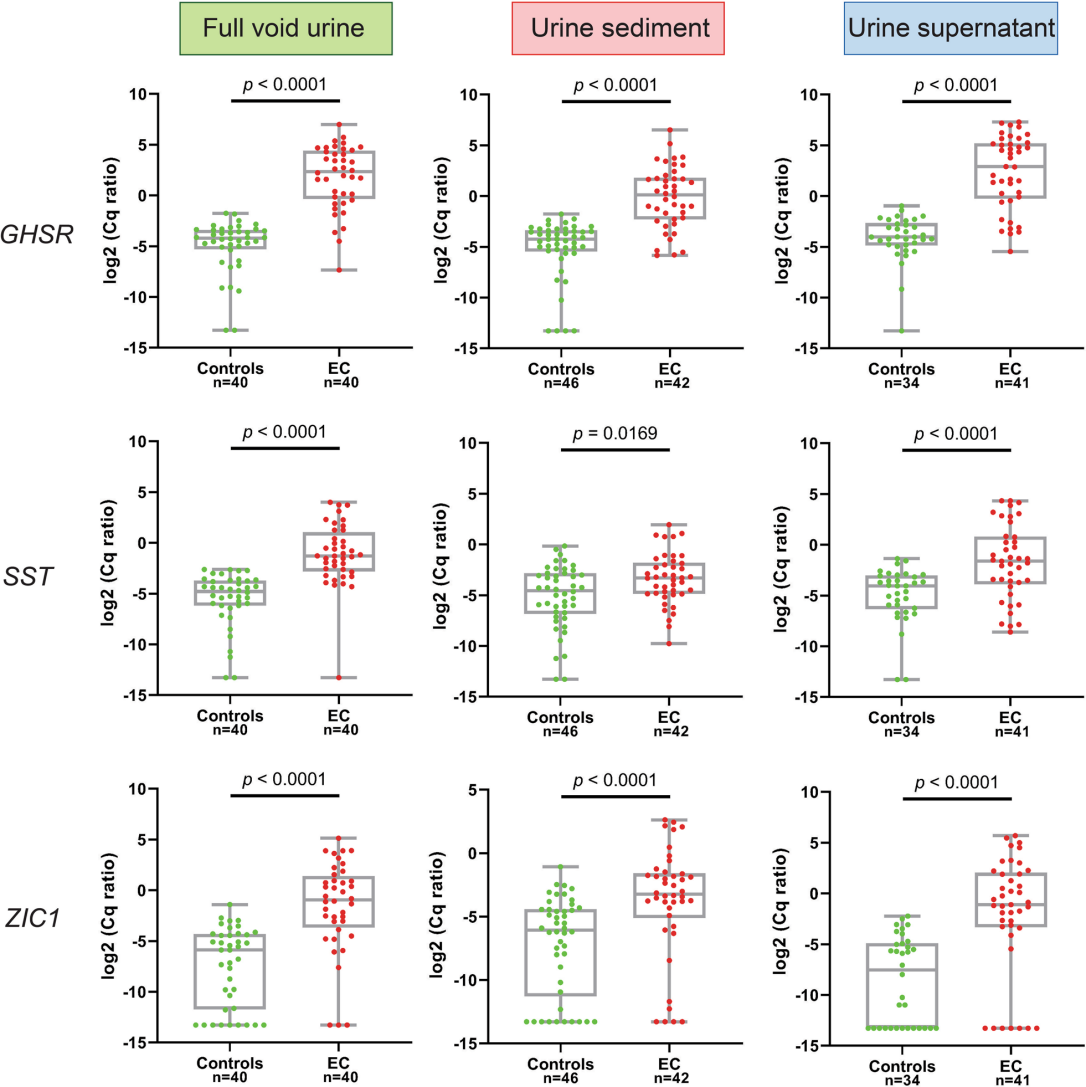
DNA methylation levels of *GHSR*, *SST*, and *ZIC1* in full void urine, urine sediment and urine supernatant from healthy female controls and women with endometrial cancer (EC)

All DNA methylation markers showed highly increased methylation levels in patients as compared to controls, resulting in *p* values < 0.001 for *GHSR* and *ZIC1* in all urine fractions, and for *SST* in full void urine and urine supernatant (Fig. 1). The diagnostic potential of each urine fraction was determined by computing ROC curves (Additional file 1: Figure S1) and quantifying AUCs of all markers (Table 4). Full void urine samples showed the highest discriminatory power for distinguishing patients from controls, with AUCs of 0.95, 0.92, and 0.86 for *GHSR, SST,* and *ZIC1*, respectively.

**Table 4:** The AUC (95% CI) of *GHSR, SST* and *ZIC1* in urine fractions for EC detection

	Full void urine	Urine sediment	Urine supernatant
*GHSR*	0.95 (0.90–1.00)	0.89 (0.81–0.96)	0.92 (0.86–0.98)
*SST*	0.92 (0.86–0.98)	0.65 (0.53–0.76)	0.76 (0.65–0.87)
*ZIC1*	0.86 (0.77–0.94)	0.76 (0.66–0.87)	0.84 (0.74–0.93)

AUC: area under the ROC curve; 95% CI: 95% confidence interval; EC: endometrial cancer

## Discussion

Urine is a promising alternative for the non-invasive detection of EC. The results of this feasibility study are the first to demonstrate that EC can be detected in urine by DNA methylation analysis with high diagnostic accuracy. A systematic comparison of different urine fractions demonstrated that full void urine is most optimal for EC detection. DNA methylation analysis of *GHSR*, *SST*, and *ZIC1* in full void urine all showed an excellent discriminatory power for EC detection (AUC 0.86–0.95).

Detecting EC in urine represents an accessible method for cancer diagnosis. The collection of urine can be done in an outpatient setting or by self-sampling at home, and can easily be performed repeatedly. Moreover, urine appears to be a stable medium for the preservation of genetic material, when handled correctly [35–37]. This enables delivery to a testing laboratory per mail.

Urine consists of a heterogeneous collection of cell components. We evaluated three urine fractions (full void, sediment, and supernatant) to determine the most optimal source of DNA for EC detection by methylation analysis*,* assuming that the urine supernatant mainly contains cell-free DNA fragments, and the urine sediment largely consists of cellular DNA [19]. Despite this supposed varying origin of DNA in the different urine components, DNA methylation analysis showed significantly increased methylation levels of all markers in all urine fractions of EC patients as compared to controls. Different urine fractions showed strong to very strong correlations (*r* ≥ 0.77–0.92). Similar findings have been described for the detection of cervical cancer [22, 23] and bladder cancer [25] in different urine fractions. When comparing the AUC values of all fractions, full void urine shows the highest potential for EC detection. An advantage of using full void urine, instead of urine sediment or urine supernatant, is that this fraction does not require pre-processing of the urine sample.

Current routine EC diagnostics are facing several challenges and limitations for which urine could offer a potential solution. Transvaginal sonography remains insufficient in distinguishing benign and malignant endometrial lesions, with a specificity that ranges from 36 to 68% among symptomatic women [38]. Apart from its limited specificity, not all endometrial malignancies present with thickened endometrium [39, 40], and the optimal cut-off of endometrial thickness that demands further examination is still under debate [41–43]. As a result, many women undergo invasive endometrial tissue sampling. This biopsy procedure can be hampered by conditions that hinder access to the uterus (e.g., cervical stenosis or discomfort) or may yield insufficient tissue for diagnosis [44].

Urine testing could not only reduce the need for performing invasive biopsies, but also has potential in screening of asymptomatic women or to triage women presenting with postmenopausal bleeding symptoms. Additionally, accurate DNA methylation marker testing in urine could be useful to monitor women with increased EC risk (e.g., women with Lynch syndrome). Among women at risk of developing EC, serial sampling of urine may offer an alternative for repeated invasive testing. Urine sampling for EC detection may also be valuable in developing countries with limited access to effective screening programs and early detection methods.

These encouraging results warrant further research to determine whether DNA methylation testing in urine meets the requirements for consideration as a diagnostic tool applicable to clinical practice in the management of EC. Currently, our sample size is being extended, together with paired cervicovaginal self-samples and clinician collected cervical scrapes to compare the diagnostic potential of DNA methylation analysis for EC detection in different sample types. We expect that a combination of present methylation markers with EC-specific markers could improve urine-based EC detection even further [33]. Since EC is more common in older women with abnormal bleeding symptoms, it is important to note that the control subjects used in this study were slightly younger and information concerning abnormal bleeding symptoms was not documented. Therefore, the specificity of this approach remains to be determined in larger source populations that also include symptomatic and asymptomatic women at risk of EC, and women with benign endometrial lesions.

## Conclusions

Our study demonstrates the feasibility of urine as a promising non-invasive specimen for EC detection. DNA methylation testing in urine could provide an attractive strategy for non-invasive EC detection for initial diagnosis during screening of asymptomatic women, to distinguish the minority of women presenting with postmenopausal bleeding symptoms due to underlying malignancy from those without EC, and to monitor women with an increased EC risk.

## Methods

### Study population

A total of 88 urine samples were used in this study, consecutively collected from women with EC (*n* = 42) and healthy female controls (*n* = 46). EC patients were recruited within the SOLUTION1 study which involved the collection of cervicovaginal and urine samples of women diagnosed with gynecological cancer. Samples from healthy female controls were collected through the Urine Controls (URIC) Biobank. Informed consent was acquired from each participating individual before urine collection. Ethical approval was obtained by the Medical Ethical Committee of the VU University Medical Center for both the SOLUTION1 study (No. 2016.213) and the use of the URIC biobank (No. 2017.112).

Enrolled patients included women with histologically proven EC of any stage before receiving primary treatment. The revised American Joint Committee on Cancer/Union for International Cancer Control Tumor-Node-Metastasis (TNM) Cancer Staging classification was used to determine tumor stage [45]. Other patient characteristics that were documented included age, histological grade and EC type. Control urine samples were retrieved from the URIC biobank (*n* = 36), including healthy volunteers without any cancer diagnosis in the past 15 years, and from our previously published healthy control cohort (*n* = 10) [22].

### Urine collection and processing

Both patients and controls collected urine at home in three 30-mL collection tubes, containing 2 mL 0.6 M ethylenediaminetetraacetic acid (EDTA) as a preservative agent (final concentration of 40 mM). Urine samples were shipped to the pathology department of Amsterdam UMC, VU University Medical Center, by regular mail and processed within 24–72 h after collection. 15 mL of full void urine was centrifuged at 3000×*g* for 15 min to separate the urine sample into two fractions: the sediment and the supernatant. The urine sediment, urine supernatant, and remaining full void urine were stored at − 20 °C. This collection and storage protocol has previously been validated for reliable DNA methylation detection in urine [36].

### DNA extraction and bisulfite modification

DNA was extracted and modified from full void urine, urine sediment and urine supernatant as described before [22, 23]. Briefly, DNA was isolated from full void urine (15 mL) and urine supernatant (15 mL) using the Quick DNA urine kit (Zymo Research, Irvine, CA, US). DNA was isolated from the urine sediment (15 mL original volume) using the DNA mini and blood mini kit (Qiagen, Hilden, Germany). DNA concentration and DNA quality were measured using a NanoDrop 1000 (ThermoFisher Scientific, Waltham, MA, US). Purified DNA was subjected to bisulfite conversion using the EZ DNA Methylation Kit (Zymo Research). All procedures were carried out according to the manufacturer’s guidelines.

### DNA methylation analysis by quantitative methylation-specific PCR (qMSP)

DNA methylation analysis of *GHSR*, *SST,* and *ZIC1* was executed by multiplex qMSP, including *ACTB*, using 50 ng modified DNA input on an ABI-7500 real-time PCR-system (Applied Biosystems, Waltham, MA, US), as described previously [22, 46]. *ACTB* was used as a reference gene for quantification and quality assessment. Sample quality was ensured by excluding samples with a quantification cycle (Cq) value exceeding 32 from methylation analysis.

### Data analysis

The DNA quality of each urine fraction of both patients and controls, of which all paired fractions were available, was examined by comparing their median *ACTB* Cq values using the Friedman test, followed by the nonparametric Wilcoxon signed-rank test. In addition, the number of samples tested invalid (i.e., excluded due to an *ACTB* Cq value ≥ 32) was documented per urine fraction.

The correlation between Cq ratios of each DNA methylation marker between paired urine fractions of both patients and controls was assessed using Spearman’s rank correlation. Correlation coefficient *r* was defined as moderate (*r* = 0.40–0.59), strong (*r* = 0.60–0.79), or very strong (*r* = 0.80–1.00).

Differences in DNA methylation levels amongst each urine fraction (i.e., full void urine, urine sediment, and urine supernatant), and between patients and controls were evaluated by comparing the log2-transformed Cq ratios. Cq ratios were computed by normalizing the methylation levels of all markers according to the reference gene *ACTB* using the comparative Cq method (2^−ΔCq^ × 100). Methylation levels of all urine fractions of both patients and controls were displayed in boxplots and tested for statistical significance using the nonparametric Mann–Whitney *U* test.

The diagnostic potential of *GHSR*, *SST*, and *ZIC1* for distinguishing patients and controls were evaluated by computing receiver operating characteristic (ROC) curves of all methylation markers, and results were quantified by the area under the curve (AUC).

Statistical analysis was performed in IBM SPSS 26, and graphs were created using GraphPad Prism 8.

## Supplementary information

**Additional file 1**: Figure S1: Receiver operating characteristic (ROC) curves of DNA methylation markers *GHSR*, *SST*, and *ZIC1* in full void urine, urine sediment, and urine supernatant. Results are quantified for all markers by an area under the curve (AUC) value.  

## Data Availability

All data generated or analyzed during this study are available from the corresponding author on reasonable request.
