# Supplementary figures and images for: Non-invasive detection of endometrial cancer by DNA methylation analysis in urine
Source: Clin Epigenetics. 2020 Nov 3;12:165. doi: 10.1186/s13148-020-00958-7 (PMC7640380; doi:10.1186/s13148-020-00958-7)

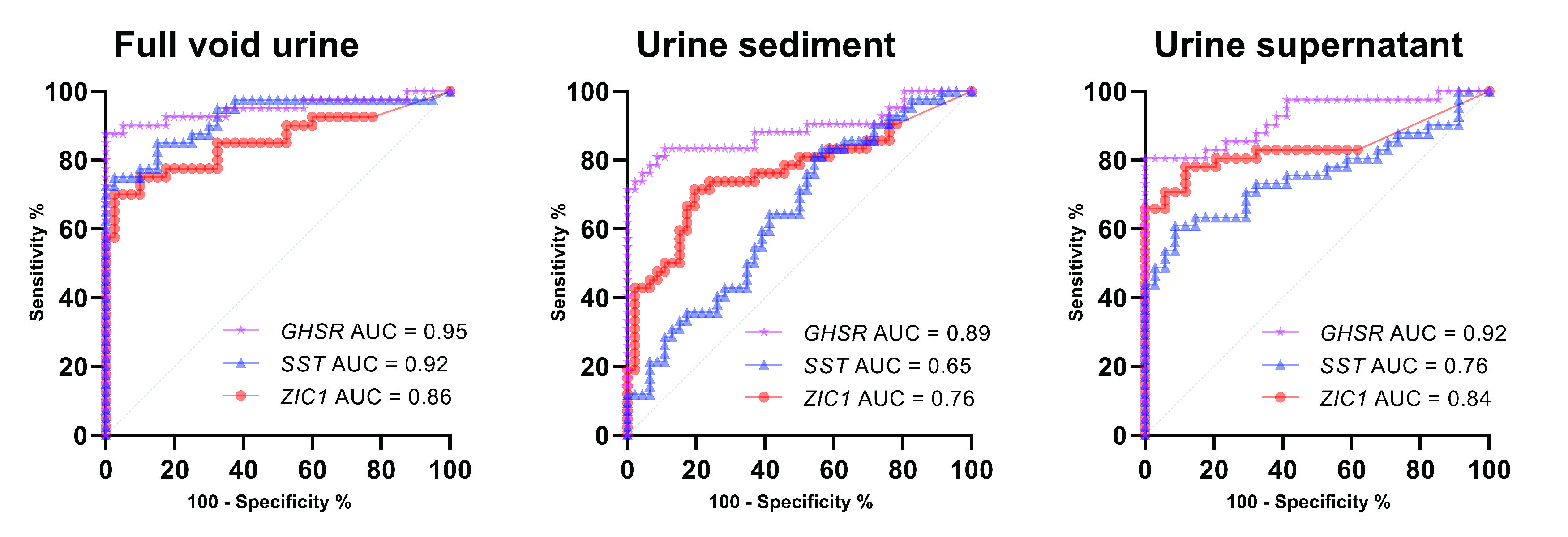

Supplement: Supplementary file 1 — Additional file 1: Figure S1: Receiver operating characteristic (ROC) curves of DNA methylation markers GHSR, SST, and ZIC1 in full void urine, urine sediment, and urine supernatant. Results are quantified for all markers by an area under the curve (AUC) value. [file 13148_2020_958_MOESM1_ESM.jpg]
